# A Patient with SIADH, Urinary Retention, Constipation, and Bell's Palsy following a Tick Bite

**DOI:** 10.1155/2022/5937131

**Published:** 2022-07-11

**Authors:** Mariana Leone, Anwar Iqbal, J. R. Hugo Bonatti, Samina Anwar, Catherine Feaga

**Affiliations:** Meritus Health, Hagerstown, MD, USA

## Abstract

*Introduction*. Lyme disease is the most common vector borne disease in the USA caused by the bacterium *Borrelia burgdorferi.* If untreated, Lyme disease can cause a variety of secondary symptoms often difficult to interpret. Some of the rare manifestations of Lyme disease include SIADH-like syndrome, enteroparesis, and urinary retention. *Case Report*. A 69-year-old male presented with anorexia and constipation and was found to have hyponatremia. Several days after admission, Bell's palsy developed and he experienced urinary retention requiring catheterization. Lyme disease was confirmed on serology, and he recalled a rash on his elbow four weeks prior. Ceftriaxone was started and the patient improved; he had multiple bowel movements after receiving laxatives and the Foley catheter was removed; serum sodium normalized with fluid restriction. He was discharged home and was well with symptoms completely resolved at three-month follow-up. *Discussion*. There should be a high alert of atypical presentation of this common tick bite associated infection. Review of the literature revealed ten similar cases, but only three of these patients were reported to have a combination of SIADH, urinary retention, and enteroparesis.

## 1. Introduction

Lyme disease is the most common vector borne disease and is caused by bacterium *Borrelia burgdorferi* (and rarely *Borrelia mayonii*) [1]. Approximately 30000 cases are annually reported to the Centers of Disease Control (CDC); it is estimated that up to almost 500000 individuals acquire the infection every year, with Maryland and Southern Pennsylvania being hotspots [1]. Lyme disease may progress through three stages, characterized as early localized stage, early disseminated stage, and late disseminated stage. Untreated Lyme disease may be complicated by chronic manifestations such as Lyme arthritis, Lyme neuroborreliosis, or other neurological manifestations [1–3]. Peripheral neuropathy is one of the common long-term complications, but involvement of the autonomic nerve system has been rarely reported. Single symptom manifestations may be more common, but subsets of patients may present with an obscure mix of symptoms delaying the diagnosis. Enteroparesis causing constipation and ileus and urinary retention are extremely rare conditions associated with Lyme disease; only a few cases of SIADH have been reported [4–9]. We herein present a patient who developed an unusual combination of symptoms, which resolved after treatment of his Lyme disease was initiated. In addition, a review of the English literature with regard to enteroparesis, urinary retention, and SIADH associated with Lyme disease was completed.

## 2. Case Report

**Figure 1:**
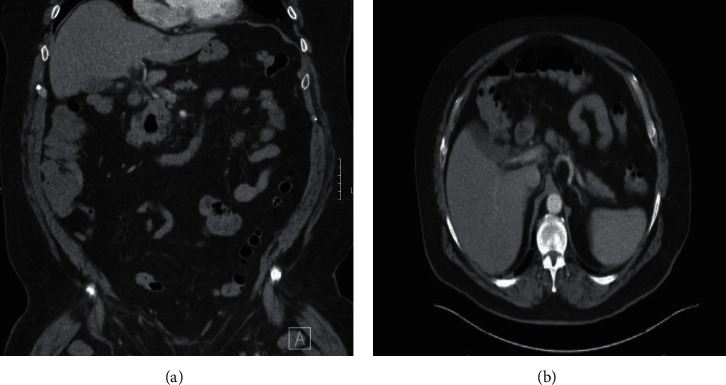
(a), (b) CT scan at initial presentation: moderate stool burden, no other findings.

A 69-year-old male with a medical history of hypertension and hyperlipidemia was initially admitted for abdominal pain and hyponatremia. He had developed decreased appetite, anorexia, and abdominal “fullness” four weeks earlier. At the same time, he experienced chills, headache, fatigue, and myalgias and noted an erythematous and edematous rash on his left elbow, which spontaneously resolved within one week. He complained of acute onset of significant constipation with no issues with moving his bowels previously and laxatives did not help. In the ER, he complained of significant abdominal discomfort. On examination, his abdomen was distended and moderately tender. WBC and CRP were normal, but sedimentation rate was elevated. He was hyponatremic with a serum sodium of 125.6 mmol/dL and potassium was 2.7 mmol/dL. Abdominal CT scan revealed no acute process but noted a moderate stool burden (Figures 1(a) and 1(b)). The patient was admitted for electrolyte correction and pain control and was started on a bowel regimen, and further work up of the obscure condition was initiated. He tested negative for SARS-COVID-19 on PCR.

Four days after his admission, the patient developed left lid ptosis and right lip numbness. He tested negative for CMV and HSV, but Lyme serology was found to be positive (total IgM and IgG antibodies). A lumbar puncture was performed. CSF was cloudy and revealed mild leukocytosis (WBC 53/microL, 45 mononucelar cells) with elevation in protein (278 mg/dl) and low glucose of 38 mg/dl (blood glucose of 95 mg/dl). Antibiotic therapy with intravenous ceftriaxone (2 g daily) was started on day 5 of his hospitalization. Late afternoon on day 7^th^ of his hospitalization (corresponding to day 2 of antibiotic initiation), the patient was found to be tachycardic, weak, diaphoretic, and febrile. Serial troponins were elevated from 3.60 ng/ml to 4.70 ng/ml, and an EKG was performed which revealed no ST segment changes, no heart block, or arrythmia, and an echocardiogram showed no acute dysfunction, no valvular pathology, and an ejection fraction of 50%. These results suggested that Lyme carditis and NSTEMI were unlikely, and that these symptoms could be a Jarisch–Herxheimer reaction. Ceftriaxone was stopped and he was switched to IV doxycycline at a dose of 100 mg bid—a bacteriostatic agent seemed the better choice over a bactericidal antibiotic. The patient's acute symptoms improved within 24 hours. Serum sodium increased to >130 mmol/dL within 5 days but then dropped again to 122 mmol/dL.

On day 13^th^ of hospitalization, the patient also began experiencing urinary retention; a Foley catheter was placed and Flomax (tamsulosin) was administered. The catheter remained in place for 72 hours and was then removed per hospital protocol, but voiding trials were unsuccessful. The Foley catheter was reinserted on day 16^th^ of hospitalization. The hyponatremia also persisted, with sodium levels ranging from 122 mmol/L to 135 mmol/L. Diagnosis of SIADH secondary to Lyme disease was supported by serum osmolality of 261 mosmol/kg, TSH of 3.96 microIU/ml, and urine studies (osmolality 650 mosmol/kg, urine sodium 101 mmol/L). With fluid restriction, hyponatremia stepwise resolved. On day 17^th^ of hospitalization, the patient was transitioned to oral doxycycline and was subsequently discharged to inpatient rehabilitation services where he remained for another 17 days with resolved urinary retention, nerve palsy and constipation, and normalized serum sodium and potassium. He was well and symptom free at the three month follow-up visit.

## 3. Discussion

Our case emphasizes that Lyme disease is capable of causing a mix of symptoms, which may be difficult to interpret, leading to a delay in diagnosis. Of note, these various symptoms may develop consecutively further obscuring the clinical picture. However, once the correct diagnosis was established and appropriate treatment was initiated, all symptoms resolved and the patient completely recovered.

The clinical manifestations of Lyme disease vary dependent on the stage of disease progression. Erythema migrans develops in approximately 50–80% of adults, and it is not uncommon that patients present with fever, malaise, fatigue, generalized pain, headaches, and other “flu-like” symptoms, during spirochete dissemination [1, 10].

Lyme disease may involve the central and less commonly the peripheral nervous system [10] with cranial neuritis most commonly involving the facial nerve, presenting with acute facial paralysis. Radiculoneuritis involving one or several dermatomes is another painful complication, and this may be accompanied by muscle weakness [10]. On rare occasions, Lyme disease may cause chronic epigastric pain [6] as well as chronic diarrhea and ulcerative colitis [11] possibly accompanied by anxiety [6, 11].

In our patient, an extremely rare mix of symptoms including SIADH, urinary retention, and constipation together with facial neuritis was observed. In our review of the literature of ten similar cases, only three patients with such a symptom complex were found, but none had facial palsy.  Table 1 provides the results of the literature review and our case. Eight reports were from the USA and three from Europe (France, Denmark, Italy). There were six men and five women with a median age of 64 (range 25–84) years. Seven patients had SIADH, six had enteroparesis, and seven had urinary retention, and all except one had additional neurologic symptoms with weakness being the most common presentation.

Most patients were treated with fluid restriction, and once the antibiotic treatment of Lyme disease was initiated, SIADH and the associated profound hyponatremia resolved [5, 7, 9, 12, 13]. SIADH may be an underreported manifestation of neuroinvasive Lyme disease.

Urinary retention was in most cases treated with catheterization, and several patients were given an alpha-adrenergic receptor antagonist. Olivares et al. were the first to report in 1995 a case of acute transverse myelitis related to Lyme neuroborreliosis leading to isolated acute urinary retention without lower-extremity impairment [14]. In our patient, transverse myelitis was suspected too, but MRI excluded this condition. Dumic et al. in 2019 reported a healthy 25-year-old man presenting with secondary erythema migrans, aseptic meningitis, and transverse myelitis causing bilateral lower-extremity motor and sensory deficits associated with urinary retention and constipation [15]. An additional four cases of enteroparesis causing constipation have been documented. Two of them were summarized by Shamin in 2005, both associated with other symptoms including hyponatremia, acute idiopathic polyneuritis with sensory deficits, constipation, and urinary retention, and patients also reported visual hallucinations [13]. Various gastrointestinal symptoms have been reported in Lyme disease, but enteroparesis is a rare symptom [13, 16]. The spectrum includes slow gastric emptying, constipation, and even acute and chronic intestinal pseudoobstruction [4, 8, 13, 17].

Our case highlights the importance of including neurological Lyme disease as a possible diagnosis in individuals who present with symptoms of autonomic dysregulation such as hyponatremia due to an SIADH-like syndrome, urinary retention, anorexia, constipation, and facial palsy.

## Figures and Tables

**Table 1:** Demographic and clinical data from review of the literature and our case.

Author	Year	Origin	Age	M/F	SIADH	Enteroparesis	Urinary retention	*n* symptoms	Other neurologic symptoms	Antibiotic treatment	Additional treatment	Outcome
Olivares	1995	France	54	M	No	No	Yes	1	None	Ceftriaxone to doxycycline	Alpha blocker, intermittent catheterization	Complete recovery
Chatila	1998	USA	61	M	No	Yes	No	1	Bells play	Doxycycline	Prednisone taper	Complete recovery
Shamim	2005	USA	64	M	Yes	Yes	Yes	3	Weakness, fatigue, hallucinations	Ceftriaxone	Fluid restriction	Complete recovery
Shamim	2005	USA	84	M	Yes	Yes	Yes	3	Weakness	Ceftriaxone	Fluid restriction, laxatives, physical therapy	Complete recovery
Perkins	2006	USA	73	F	Yes	No	No	1	Unsteady gait, falls, decreased mental activity, daytime somnolence	Ceftriaxone	Fluid restriction	Complete recovery
Schefte	2015	Denmark	66	F	No	Yes	Yes	2	Lower back pain	Ceftriaxone	Laxatives	Complete recovery
Siddiqui	2017	USA	83	F	Yes	No	No	1	Bilateral arm weakness	Ceftriaxone	Fluid restriction, salt tablets	Complete recovery
Dumic	2018	USA	25	M	No	No	Yes	1	Frequent falls, bilateral lower-extremity weakness and numbness	Ceftriaxone	Steroid taper, acyclovir, intermittent catheterization	Slow recovery
Saalami	2018	USA	62	F	Yes	Yes	Yes	3	Lower back pain, weakness	Ceftriaxone	Hypertonic saline, fluid restriction	Complete recovery
Da Porto	2019	Italy	62	M	Yes	No	No	1	Weakness, dizziness, confusion, backpain	Ceftriaxone	NS infusion, fluid restriction	Slow recovery
Leone	2020	USA	69	M	Yes	Yes	Yes	3	Bell's palsy, anorexia	Ceftriaxone to doxycycline	Fluid restriction, foley catheter, alpha blocker	Complete recovery
